# An Integrated Kinematic Modeling and Experimental Approach for an Active Endoscope

**DOI:** 10.3389/frobt.2021.667205

**Published:** 2021-06-28

**Authors:** Andrew Isbister, Nicola Y. Bailey, Ioannis Georgilas

**Affiliations:** Department of Mechanical Engineering, University of Bath, Bath, United Kingdom

**Keywords:** endoscopic robots, experimental validation, Cosserat theory, closed-loop control, actuation

## Abstract

Continuum robots are a type of robotic device that are characterized by their flexibility and dexterity, thus making them ideal for an active endoscope. Instead of articulated joints they have flexible backbones that can be manipulated remotely, usually through tendons secured onto structures attached to the backbone. This structure makes them lightweight and ideal to be miniaturized for endoscopic applications. However, their flexibility poses technical challenges in the modeling and control of these devices, especially when closed-loop control is needed, as is the case in medical applications. There are two main approaches in the modeling of continuum robots, the first is to theoretically model the behavior of the backbone and the interaction with the tendons, while the second is to collect experimental observations and retrospectively apply a model that can approximate their apparent behavior. Both approaches are affected by the complexity of continuum robots through either model accuracy/computational time (theoretical method) or missing complex system interactions and lacking expandability (experimental method). In this work, theoretical and experimental descriptions of an endoscopic continuum robot are merged. A simplified yet representative mathematical model of a continuum robot is developed, in which the backbone model is based on Cosserat rod theory and is coupled to the tendon tensions. A robust numerical technique is formulated that has low computational costs. A bespoke experimental facility with precise automated motion of the backbone via the precise control of tendon tension, leads to a robust and detailed description of the system behavior provided through a contactless sensor. The resulting facility achieves a real-world mean positioning error of 3.95% of the backbone length for the examined range of tendon tensions which performs favourably to existing approaches. Moreover, it incorporates hysteresis behavior that could not be predicted by the theoretical modeling alone, reinforcing the benefits of the hybrid approach. The proposed workflow is theoretically grounded and experimentally validated allowing precise prediction of the continuum robot behavior, adhering to realistic observations. Based on this accurate estimation and the fact it is geometrically agnostic enables the proposed model to be scaled for various robotic endoscopes.

## 1 Introduction

Continuum robots are inspired by nature and enable positioning of an end effector via a backbone that bends continuously along its length Stassen et al. (2001), Robinson and Davies (1999). This is achievable as continuum robots do not contain rigid links or discrete joints, but motion actuation occurs remotely, usually by a set of cables or tendons. A further development of this in recent years has been the adoption of soft-robotics whereupon material that are intrinsically compliant are used, as in Rus and Tolley (2015).

This category of robots are characterized by their high manoeuvrability in unstructured and confined environments Walker et al. (2016), being exceptionally versatile for performing delicate tasks Cianchetti et al. (2015), and their capability to perform dexterous grasping and manipulation Giannaccini et al. (2014), Katzschmann et al. (2015). Their dexterous capabilities make them ideal in medical applications where flexibility and manipulation is necessary Burgner-Kahrs et al. (2015), Cianchetti and Menciassi (2017) including in active endoscopes.

Nonetheless, this class of robots pose a significant challenge in terms of control. The issue lies in the manner of which such a flexible structure can be described. In classical robotics the mechanisms are rigid and have well defined shapes enabling an analytical description of their kinematics. For continuum robots alternative theoretical methods have been proposed. One of the most prominent approaches is a constant curvature approach, as used by Webster and Jones (2010), which is similar to the traditional description of rigid robots; it approximates the robot as a finite series of constant-curvature arcs. Most continuum robots do not follow this behavior in reality, however, some do approximately have this behavior (for example see Webster et al., 2009). This approach has been applied to a variety of continuum robots, including tension actuated, due to it being applicable to a wide range of designs and the simplifications in the kinematic modeling. This is because the arc length parameters can be converted into analytical frame transformations. However, there are bounds on the continuum robots design and loading for the approximation of the constant curvature approach to be valid, for example see Li and Rahn (2002).

A variable curvature approach, which is also based on a geometric description, has also been applied to a continuum robot by Trivedi et al. (2008), which tries to capture the flexible nature of a continuum robot. An alternative to the geometric description of the continuum robot is to use the mechanical properties of the body. This usually takes the form of the conservation law of energy. Of the most dominant methods is the use of beam theory as used by Gravagne et al. (2003); Sadati et al. (2017), together with the Cosserat rod method implemented by Trivedi et al. (2008), Burgner-Kahrs et al. (2015). Additionally to the kinematic modeling, the deformation of the continuum robot may be identified through these methodologies, better predicting the shape of continuum robot over a range of conditions, including under external loads. However, they are generally computationally complex and non-intuitive, relative to constant curvature modeling. The aim of this work is to address the need to maintain the accuracy as much as possible while reducing the complexity and computation cost.

Additionally the continuum robot and the actuation method needs to be coupled together efficiently to give reliable results. For tendon driven continuum robots, Rucker and Webster (2011) examined the case of external point and distributed loads on the backbone from tendon tension and external forces. After the mechanism has been described the governing model needs to be numerically solved to assist with the control of the robot. The newest proposed method to so is by using a Reduced-Order-Model with the Ritz method, as used by Sadati et al. (2018), Tunay (2013), and specialized solution methods and toolboxes have been developed to this end, Sadati et al. (2019).

This work focuses on investigating a direct method of solving a continuum robot model based on Cosserat theory, chosen as it is independent of any specific discretization scheme and so is able to predict the behavior of the backbone under large deformation more accurately. A simplified approach for coupling the tendon tensions to the backbone is utilized to reduce the necessary numerical computations. A robust numerical technique is developed and bespoke code is formulated. This approach gives a compromise between the computation time and the model accuracy, however it is sufficiently precise for practical implementation with predictions representing the backbone curvature and position well. Additionally, the bespoke experimental facility is novel as it can be controlled automatically by regulating the tendon tension through motors and strain gauges to allows the backbone to be moved in a smooth and orderly motion. The model is built about the assumption that by adjusting the boundary condition within the classical Cosserat rod model to suitably account for the tendon loads which act on the continuum robots, as well as external loads. The research presented here is investigating this premise for a 3D continuum robot, with the model developed in Section 2.1 and validated for this type. The assumptions are tested using a prototype system which is actuated by four equally spaced antagonistic tendons running parallel to a backbone, as shown in Section 2.2. The results are given in Section 3 with Section 4 presenting the discussion. This paper demonstrates how the slender size of the continuum robot and the naturally slow motion of endoscopes allow the dynamics of the backbone to be assumed negligible Alqumsan et al. (2019), giving a simplified kinematic model. One of the major contributions of this work is to investigate the practical frictional characteristics of tendons moving against support structures to draw useful conclusions for the applicability of the model to scaled down versions of the continuum robot. This last step is crucial to allow for the translation of the proposed work to be applicable for endoscopic systems.

## 2 Materials and Methods

### 2.1 Model of the Flexible Backbone

A continuum robot backbone model is developed, based on the classical nonlinear Cosserat rod theory. The backbone can be described effectively by an elastic rod in three dimensions, which accounts for the nonlinearity of rod bending [see Alqumsan et al. (2019), Robinson and Davies (1999)]. This approach discounts the previously common modeling assumption of constant curvature of the backbone.

**FIGURE 1:**
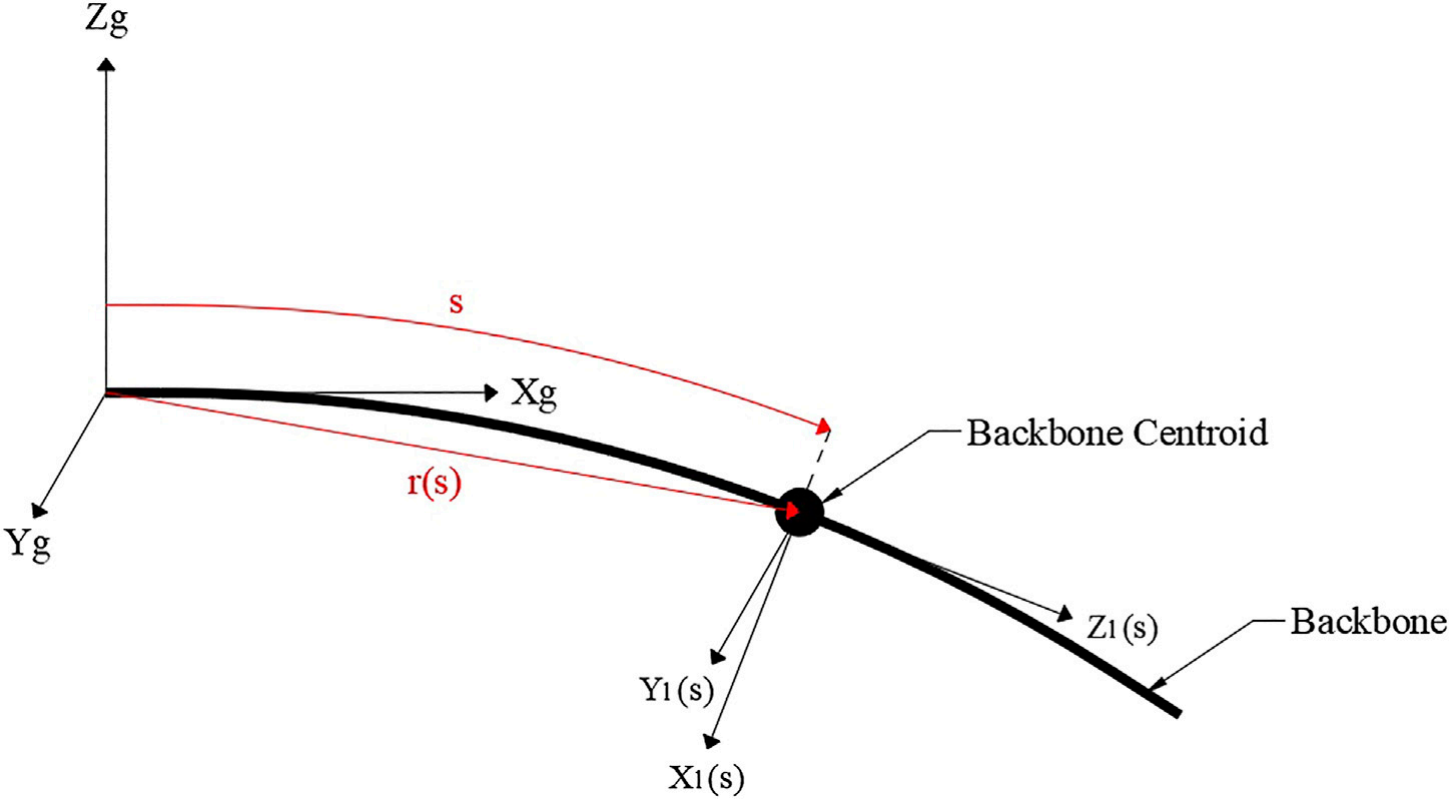
Backbone showing the backbone centroid at an arc length *s* and distance *r* from the global origin with global and local coordinate systems (Xg,Yg,Zg) and $\left(X_{1},Y_{1},Z_{1}\right)$, respectively.

The centroid position along the backbone arc length s∈[0,l] together with its local orientation with respect to the global origin and coordinate system are given by r(s) and rotation matrix R(s), respectively, as shown in Figure 1 with a Cartesian coordinate system. The backbone centroid is as depicted in Figure 1 and the local linear and angular rates of change of the backbone are denoted by u(s) and Ω(s), respectively. The corresponding equations are given as

$$\text{r}\left(s\right)=\left(\begin{array}{c} r_{x}\\ r_{y}\\ r_{z} \end{array}\right),\;\;\;\text{u}\left(s\right)=\left(\begin{array}{c} u_{x}\\ u_{y}\\ u_{z} \end{array}\right),\;\;\;\overset{⌢}{\Omega }\left(s\right)=\left(\begin{array}{ccc} 0 & -{\text{Ω}}_{z} & {\text{Ω}}_{y}\\ {\text{Ω}}_{z} & 0 & -{\text{Ω}}_{x}\\ -{\text{Ω}}_{y} & {\text{Ω}}_{x} & 0 \end{array}\right),$$

$$\text{R}\left(s\right)\;=\left(\begin{array}{ccc} \cos\phi \cos\theta & \cos\psi \sin\theta \sin\phi & \cos\psi \sin\theta \cos\phi \;+\;\sin\psi \sin\phi \\ \sin\psi \cos\theta & \sin\psi \sin\theta \sin\phi \;+\;\cos\psi \cos\phi & \sin\phi \sin\theta \cos\phi \;-\;\cos\psi \sin\phi \\ -\text{sin}\theta & \cos\theta \;\;\sin\phi & \cos\theta \;\;\cos\phi \end{array}\right),$$ (1)

where ψ, θ, and ϕ refer to the angles through which the local axes have turned about the global *x*, *y*, and *z* axes, respectively, and the hat denotes the skew matrix form. The transformation of the backbone along its arc length *s* can therefore be defined by

$$\dot{\text{r}}\left(s\right)=\text{R}\left(s\right)\text{u}\left(s\right),\;\;\;\;\;\;\;\;\;\dot{\text{R}}\left(s\right)=\text{R}\left(s\right)\overset{⌢}{\Omega }\left(s\right),$$ (2)

where R(s) acts to transform the points from a local to global coordinate system and $\dot{R}$ denotes the derivative of *R* with respect to the arc length *s*.

The backbone internal force and moments in global coordinates are given by

$$\text{n}\left(s\right)=\left(\begin{array}{c} n_{x}\\ n_{y}\\ n_{z} \end{array}\right),\;\;\;\;\;\;\;\;\;\;\text{m}\left(s\right)=\left(\begin{array}{c} m_{x}\\ m_{y}\\ m_{z} \end{array}\right).$$ (3)

The distributed load applied to the backbone f(s) is given by the negative of the derivative of n(s) with respect to *s*, which is taken to be the weight of the backbone. Similarly, due to the internal force n(s) acting at a given global coordinate r(s), as in Figure 1, the derivative of m(s) with respect to *s* can be equated to the negative of the cross product of $\dot{\text{r}}\left(s\right)$ and n(s). This results in the set of equations for the rate of change of internal forces and moments

$$\dot{\text{n}}\left(s\right)=-\text{f}\left(s\right),\;\;\;\;\;\;\;\;\;\;\dot{\text{m}}\left(s\right)=-\dot{\text{r}}\left(s\right)\times \text{n}\left(s\right).$$ (4)

The constitutive law dictates that the backbone internal forces and moments can be described by the difference between the deformed and undeformed states at arc length *s* along the backbone. The undeformed state is given by subscript * and is assumed to be a straight cylindrical rod extending in the local *z* axis, giving ${\text{u}}^{\text{*}}={\left(0,0,1\right)}^{T}$ and $\Omega^{\text{*}}={\left(0,0,0\right)}^{T}$. The internal forces and moments depend on the backbone stiffness in shear and extension for the forces and bending and torsion for the moments, characterized by matrices $K_{se}$ and $K_{bt}$, respectively;

$$\text{n}\left(s\right)=\text{R}\left(s\right){\text{K}}_{se}\left(\text{u}\left(s\right)-{\text{u}}^{\text{*}}\right),\;\;\;\;\;\;\;\;\;\;\text{m}\left(s\right)=\text{R}\left(s\right){\text{K}}_{bt}\left(\Omega \left(s\right)-\Omega^{\text{*}}\right),$$ (5)

where

$${\text{K}}_{se}\left(s\right)=\left(\begin{array}{ccc} GA & 0 & 0\\ 0 & GA & 0\\ 0 & 0 & EA \end{array}\right),{\text{K}}_{bt}\left(s\right)=\left(\begin{array}{ccc} EI_{x} & 0 & 0\\ 0 & EI_{y} & 0\\ 0 & 0 & EI_{z} \end{array}\right),{\text{u}}^{\text{*}}=\left(\begin{array}{c} 0\\ 0\\ 1 \end{array}\right),\Omega^{\text{*}}=\left(\begin{array}{c} 0\\ 0\\ 0 \end{array}\right).$$ (6)

*G* denotes the shear modulus, *A* the cross sectional area of the backbone, *E* the Young’s Modulus and *I* the second moment of area about the subscript coordinate.

Therefore, the set of governing equations for the global coordinates, formed from Eqs. 2–5, will be

$$\dot{\text{r}}\left(s\right)=\text{R}\left(s\right)\text{u}\left(s\right),\;\;\;\;\;\;\;\;\;\;\dot{\text{R}}\left(s\right)=\text{R}\left(s\right)\hat{\Omega }\left(s\right),\;\;\;\;\;\;\;\;\;\;\dot{\text{n}}\left(s\right)=-\text{f}\left(s\right),\;\;\;\;\;\;\;\;\;\;\text{m}\left(s\right)=-\dot{\text{r}}\left(s\right)\times \text{n}\left(s\right),$$ (7)

with boundary conditions

$$\text{r}\left(0\right)=\left(0,0,0\right),\;\;\;\;\;\;\;\;\;\;\text{R}\left(0\right)=\left(\begin{array}{ccc} 0 & 0 & 0\\ 0 & 0 & 0\\ 0 & 0 & 0 \end{array}\right),\text{n}\left(l\right)=\text{F},\;\;\;\;\;\;\;\;\;\;\text{m}\left(l\right)=\text{L},$$ (8)

where F and L are the forces and moments applied at the backbone tip, respectively. The local coordinates are computed using

$$\text{u}\left(s\right)={\text{K}}_{se}^{-1}{\text{R}}^{\text{T}}\left(s\right)\text{n}\left(s\right)+{\text{u}}^{\text{*}},\;\;\;\;\;\;\;\;\;\;\Omega \left(s\right)={\text{K}}_{bt}^{-1}{\text{R}}^{\text{T}}\left(s\right)\text{m}\left(s\right)+\Omega^{\text{*}}.$$ (9)

The set of governing Eq. 7 with corresponding boundary conditions 8) are solved simultaneously using a boundary value problem solver based on a finite difference code that implements the four-stage Lobatto IIIa formula which has high mathematical efficiency and robustness (see Shampine and Kierzenka (2008) for details).

### 2.2 Experimental Set-up

**FIGURE 2:**
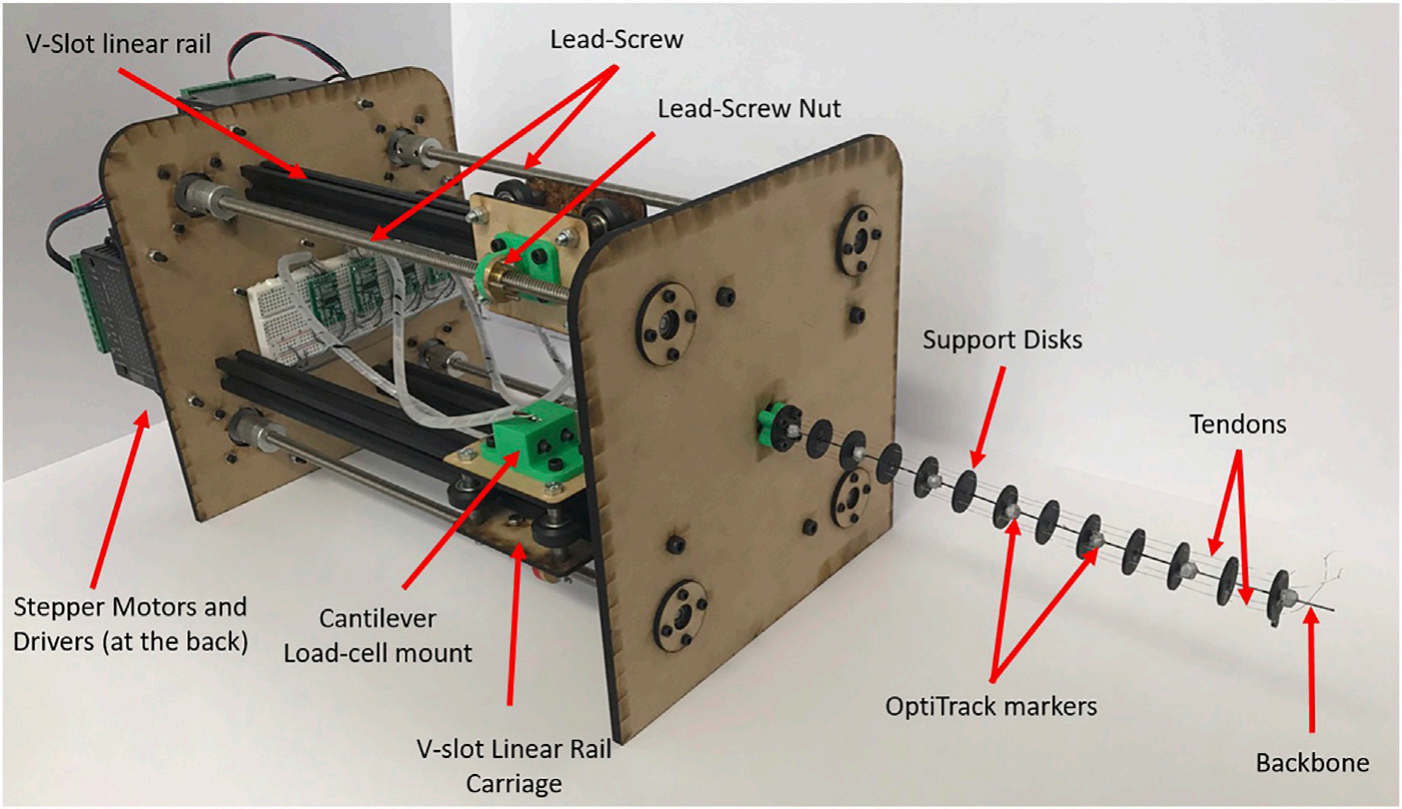
Constructed continuum robot rig with backbone, actuated by tendons running through support disks by stepper motor and lead screws. Cantilever load-cells measure the tendon tension and OptiTrack markers are included for backbone position detection.

The experimental set-up for evaluating the model can be seen in Figure 2. The backbone is made of ASTMA228 spring steel and has a length of 240 mm, chosen such that large curvature can be achieved without plastic deformations. Four 0.28 mm 4-strands Dyneema (Dingbear, China) tendons are chosen to minimize elongation of the tendon itself while providing high fracture stress. The tendons are numbered 1–4 in a clockwise direction, when looking end on, starting from the middle right tendon, giving tendons 1 and 3 on the horizontal plane, and tendons 2 and 4 on the vertical plane, see Figure 6.

Acrylic circular support discs were used to minimize friction between the tendon and support discs; additionally Teflon spray was used to further reduce friction. A total of 13 support discs of 20 mm diameter and 1.5 mm thickness were used and spaced 20 mm apart along the backbone, such that an optimum ratio of support disc spacing to tendon offset from backbone of 2.5 mm was achieved as proposed by Rucker and Webster (2011). The tendons passed through the support discs at a distance of 8 mm from the backbone central axis and are equally spaced around the circle circumference.

Each tendon is actuated independently, through an assembly comprising of a lead screw mechanism driven by a stepper motor. The stepper motors have a resolution of $0.05625^{\circ }$ and the lead screw provides 0.008 m of translation per rotation, resulting in a linear resolution of $1.25\times 10^{-5}\;\mathrm{mm}$ per step. To stabilize the system and ensure linear translation of the lead crew nut, a v-slot linear rail and carriage system is used, together with its ability to translate with minimal friction which is necessary for the high tendon tension case.

A cantilever load cell is fixed to the carriage, which in turn is connected to the tendon, causing it to move through a set of forces and a moment offering a loading feedback for each of the tendons. A 1 kg load cell (Phidgets, Canada) was chosen to identify the tension as a compromise between the maximum expected tendon tension and resolution.

The position of the backbone was recorded using a contactless sensor, namely OptiTrack (Target3D, United Kingdom) motion capture system; this eliminated any error that may be associated with contact sensors. Reflective markers of 6 mm diameter were incorporated on the face of every second support disc. Seven cameras were used, to ensure uninterrupted tracking of the markers, with a maximum root mean square positional error of 0.105 mm achieved. The initial position of the backbone, before any tendon loading, was identified once a stable tension reading of the desired value ±0.01 N had been achieved.

### 2.3 Representative Model of Experimental Facility

**FIGURE 3:**
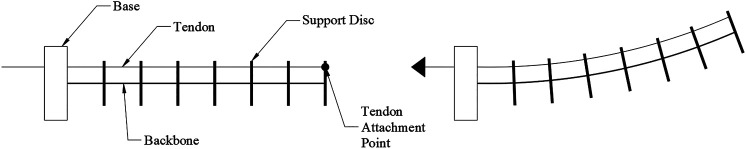
Single tendon continuum robot actuation (**left**-no tension in the tendon, **right**–tendon in tension).

The backbone model in Section 2.1 is modified to represent the experimental set-up with the tendons coupled to the backbone as shown in Figure 3 using support discs to provide actuation of the backbone; moments on the end support disk of the backbone must be included, which are denoted by $L_{i}$, where i=1−4 for tendons 1−4, respectively. The tendons also produce an axial point force at the tip location, however, these are assumed negligible as the distributed force that the tendons apply to the support discs are at least an order of magnitude larger Gravagne et al. (2003). Nevertheless, the boundary conditions can be adjusted for any externally applied end load through the internal force n(l). Therefore, by neglecting the distributed forces from the tendon onto the backbone, and only considering the moment they impose on the backbone tip, the number of numerical computations that are needed at each iteration.

When the backbone is undeformed, it is assumed moments $L_{1}$ and $L_{3}$ act around the global *z* axis and $L_{2}$ and $L_{4}$ about the *y* axis as shown by Figure 1. Each applied tendon moment is calculated using $L_{i}=T_{i}h$, where $T_{i}$ is the tendon tension on the $i^{\text{th}}$ tendon and *h* is the tendon offset from the backbone. This leads to the boundary condition for the internal moment at the tip location

$$\text{m}\left(l\right)=\text{L}=\left(\begin{array}{c} 0\\ L_{4}-L_{2}\\ L_{1}-L_{3} \end{array}\right).$$ (10)

Therefore, the boundary conditions in Eq. 8 are modified to accommodate Eq. 10.

### 2.4 Tension Closed Loop Control

**FIGURE 4:**
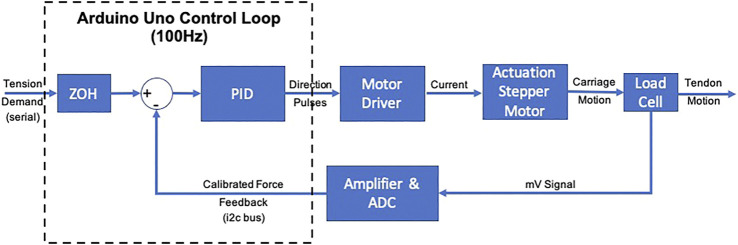
Schematic representation of the closed-loop control for tendon tensioning. The demand is calculated from the model and transmitted to the Arduino Uno and hold via a ZOH. The Control Loop runs at 100 Hz and runs a PID controller based on the error between the demand and the Force Feedback. The control signal are pulses for the Motor driver that generates the current to run the stepper motor. The lead-screw mechanism applies the tension to the tendon via the cantilever load cell. The measurements from the latter are being amplified and sampled by from the ADC at the rate of the Control Loop.

Based on the boundary conditions in Eq. 8 and representation model of experimental facility in Section 2.3, the control variable is selected to be the tension of the tendons, $T_{i}$. This firstly offers a simpler control approach and secondly ensures that any frictional effects are directly countered by the motor. The closed loop control of the tendon tension was implemented as a PID controller on an Arduino Uno (Arduino, Italy), see Figure 4. The Arduino Uno receives demand tension values from the model via serial communication from an i7-7500U, with 8 Gb memory running Matlab (Mathworks, United States) on Windows 10. The demand values are held via a zero-order hold and the control loop is run at 100 Hz. The tension feedback was received from the load cell via an integrated amplifier/ADC NAU7802 (Nuvoton, Taiwan) via an i2c bus interface, sampled at the control loop frequency.

A PID controller architecture was selected given the overall behavior of the system. Although PD is the preferred method in continuum robots George Thuruthel et al. (2018) it can be observed that PD control guarantees stability only when the PD gains tend to infinity Slotine and Li (1991). Moreover the steady state error does not tend to zero unless there is some form of model-based compensation. Since the presented model does not involve dynamic behavior and the tension controller, inclusions of the Integral term was done to ensure minimizing the steady state error by compensating tendon friction.

**FIGURE 5:**
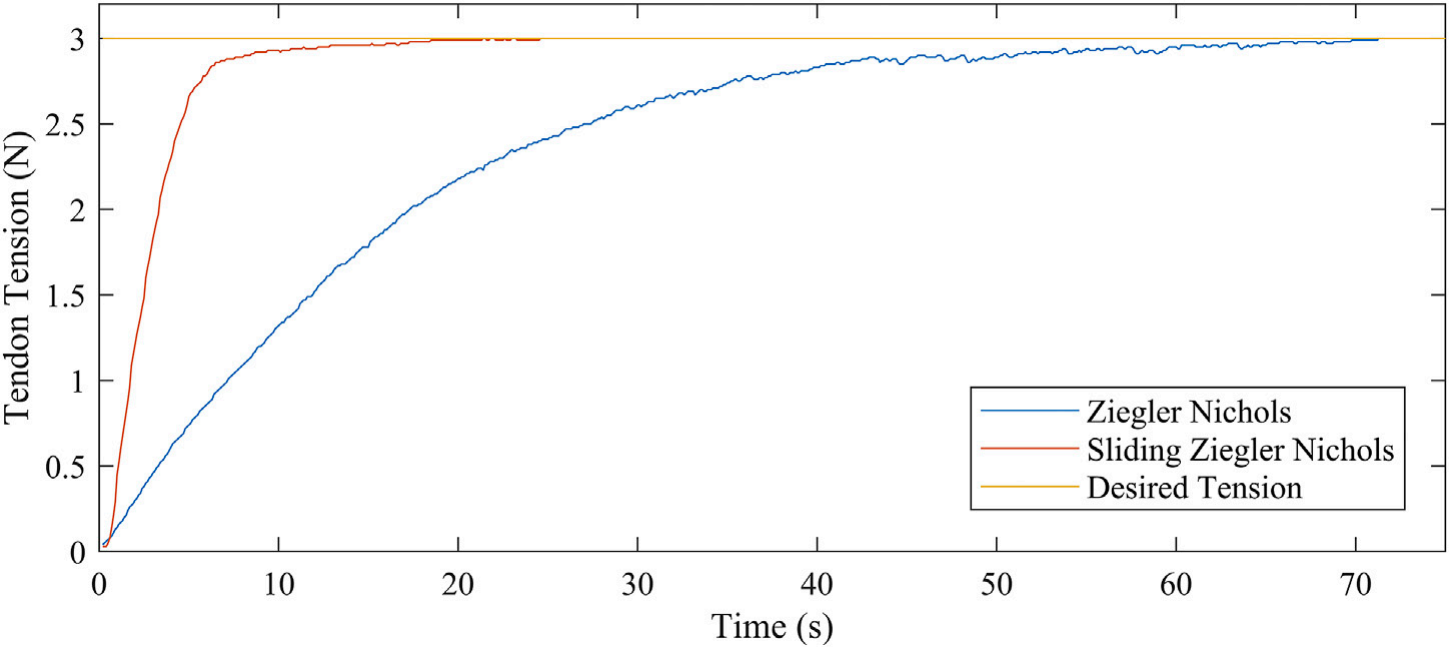
Continuum robot control curves for two PID tuning techniques showing the tendon tension against time.

Initially, the tuning procedure for the PID controller followed the classic Ziegler-Nichols method based on a step response of a single tendon being tensioned from 0 to 3 N. This procedure resulted in zero steady state error and minimal oscillations with the calculated PID gains for the proportional, integral and derivative terms of $K_{p}=0.15$, $K_{i}=3$, and $K_{d}=0.15$, respectively. However, as shown in Figure 5, it resulted in a rise time of 70 s was which was deemed insufficiently long.

Therefore, to reduce the system rise time to 25 s, a two-stage proportional controller was implemented. This is similar to a divide and conquer gain-scheduling controller Rugh and Shamma (2000) with the only evaluation criterion being the desired rise time and tendon tension. In stage one an initial high value of $K_{p}=1$ was used. This value then switched in the second-stage to the value identified by the Ziegler-Nichols method ($K_{p}=0.15$), to maintain the desired steady state characteristics. The threshold at which this switch occurred was determined to be relative to the desired tendon tension ($T_{des}$) at a value of $T_{des}\pm 0.2$ N. The integral, and derivative gains remained the same for the two control stages.

## 3 Results

A set of 15 tension combinations are used for all experimental evaluations. As tendons 1 and 3 act in the horizontal plane and tendons 2 and 4 act in the vertical plane, tendon 3 remained slack always and combinations of tendon 1 with either tendon 2 or 4 were used. This was to examine the effect of gravity, as tendons 2 and 4 acting against or with gravity, respectively. To minimize the backbone stress and enable reliable data for comparison with the model, only one tendon from each plane was tensioned. It is assumed that if tendon 1 remained slack, but combinations of tendon 3 with either tendon 2 or 4 were examined, solutions would be the same but mirrored along the central vertical position.

**FIGURE 6:**
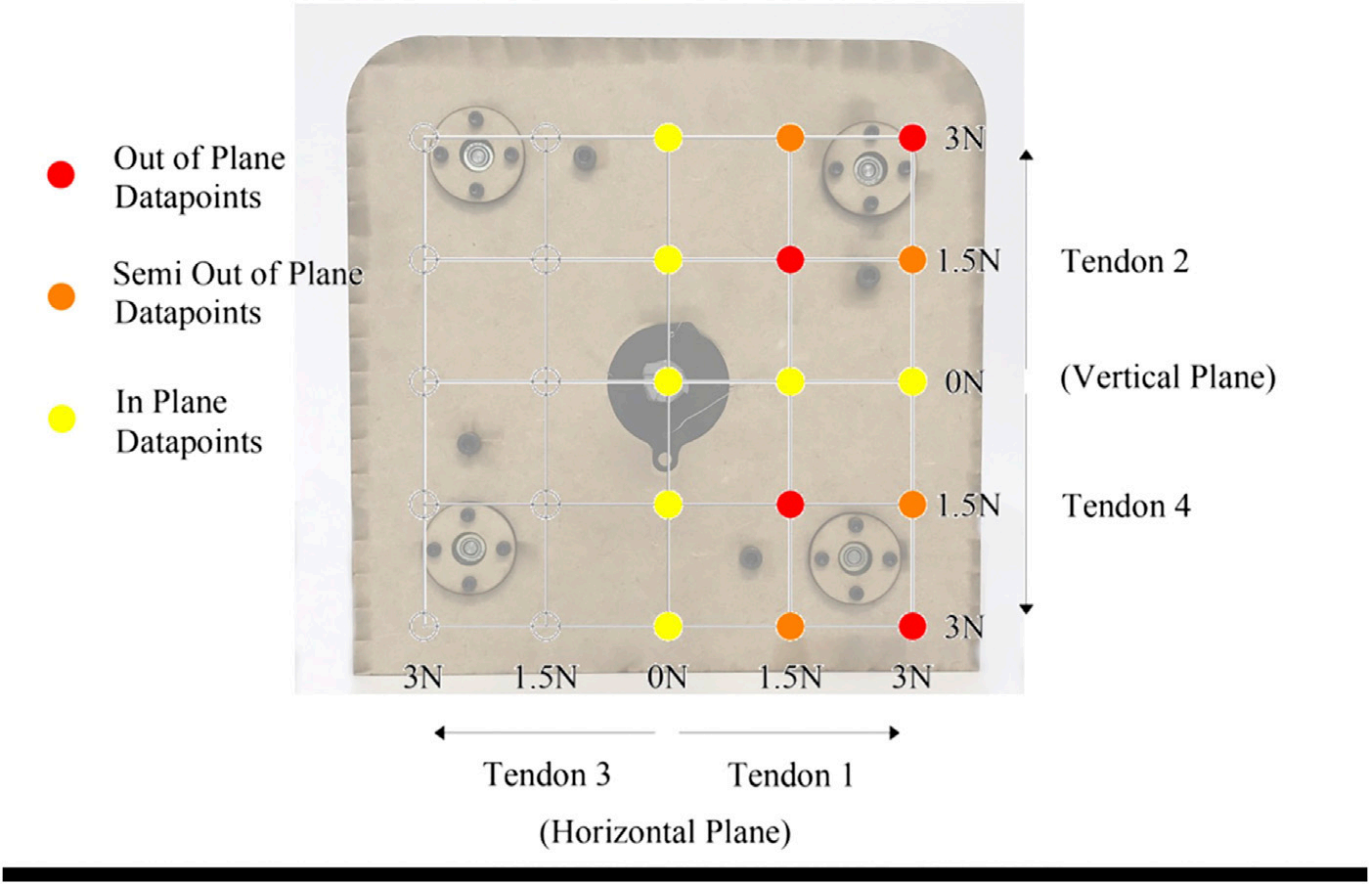
Overlay of experimental tendon tension combinations. Three different regions are defined; In-Plane, Semi-Out-of-Plane and Out-of-Plane data points.

Each tendon could be tensioned with a value of 0, 1.5 or 3 N. The combinations were divided into three groups, a) In-Plane, b) Semi-Out-of-Plane and c) Out-of-Plane which can be seen in Figure 6. In-Plane points are when at least one of the two tensions is 0 N, Semi-Out-of-Plane has a combination of tensions 1.5 and 3 N for either tensioned tendon and Out-of-Plane motion is when tension in both tendons are either 1.5 N or 3 N. These combination were selected to address the effect of weight to the loading of two of the tendons.

When conducting the experiments, the error, defined as the distance between the predicted and realized tip location, is quoted as a percentage of the backbone length, enabling comparison of the model accuracy with those approaches already available in literature.

### 3.1 Frictional Effects of Tendon-Disk Interface

In order to evaluate the validity of the results produced by the experimental set-up, the effect of tendon-disk interface friction on the system repeatability was investigated. The tension in tendons 1 and 3 were kept at 0 N while the tension in tendons 2 and 4 were changed, but the tension in only one tendon was non-zero at any given time, to a value of either 1.5 N or 3 N. Experiments were carried out where these values were achieved by increasing the tension from 0 N as well as by over tensioning the tendon to 5 N before decreasing it to the desired value which allowed for the removal of tendon slack from within the support discs. The relative distance of the backbone tip from the origin was recorded and analyzed for each repeat.

**FIGURE 7:**
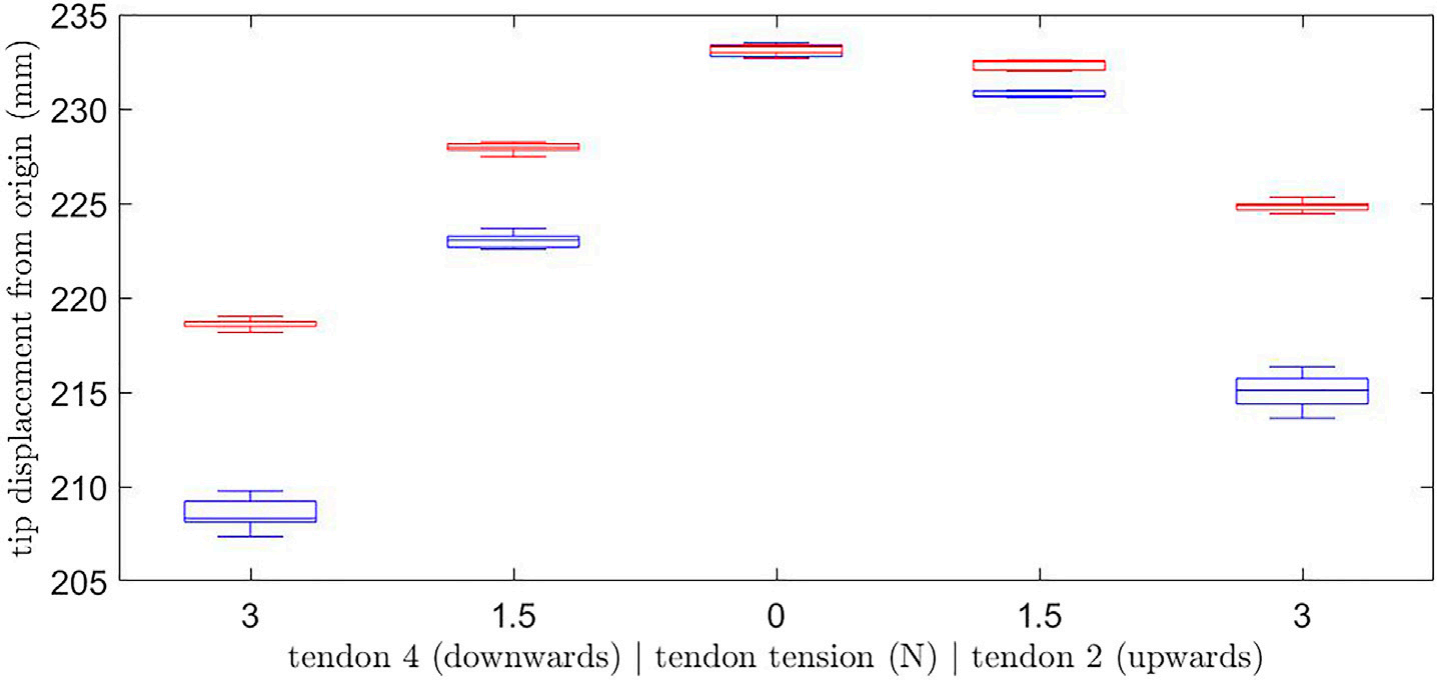
Tip displacement from the global origin for tensions of 0, 1.5 or 3 N in tendon two or 4 (only one tendon is tensioned at a time) in the vertical plane when the desired value is achieved by increasing the tension from 0 N (red) or decreasing from 5 N (blue).

The results of the relative distance of the backbone tip from the origin for different tensions in the tendons in the vertical plane are shown in Figure 7 when the desired value is achieved by increasing the tension from 0 N (red) or decreasing from 5 N (blue). Table 1 summarizes the standard deviation for each test case. From these results it can be seen that when the tension is 0 N in both tendon 2 and 4 the experimental facility is consistent in achieving the same position when either increasing or decreasing the tension to 0 N. However, for all other cases examined, when the desired tension is achieved by decreasing it from 5 N the backbone tip position is further from the fixed end at the origin than when the tension is increasing from 0 N. This indicates that the backbone has a greater amount of bending when the tension is achieved by decreasing it from 5 N for all loading cases. The difference between the two methods of tensioning the tendons increases with the tendon tension magnitude and is attributed to stiction/friction occurring between the tendon and the support discs, so that when the tension is increased from 0 N there is some slack left in the system. Given this observation the subsequent experiments will be tensioned to the desired value, then the backbone will be returned to the neutral position before performing the next force combination.

**TABLE 1:** Standard deviation of the backbone tip location for different tensions when increasing from 0 N or decreasing from 5 N.

	Vertical tendon tension (N)				
Tension (N)	Tendon 4			Tendon 2	
		3	1.5	0	1.5	3
Standard deviation when increasing from 0 N (mm)	2.94	2.22	2.60	2.18	2.59
Standard deviation when decreasing from 5 N (mm)	7.77	3.48	3.29	1.32	9.43

### 3.2 Young’s Modulus Estimation

**FIGURE 8:**
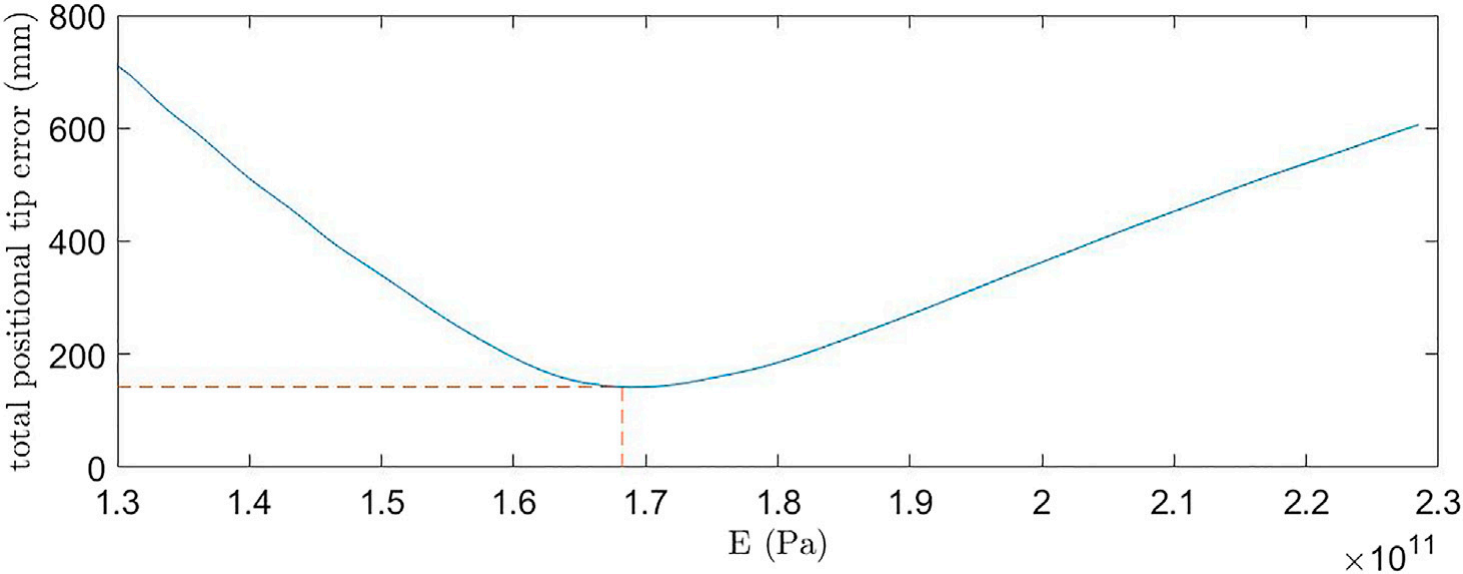
Total positional tip error between the theoretical prediction and experimental result for increasing Young’s Modulus.

The impact of the support disks and the OptiTracker markers on the modulus of elasticity of the rod is investigated, to enable model validation. Based on these measurements the updated modulus is used to perform the calculation for the model and used subsequently. To identify the modified Young’s Modulus, values of *E* were swept in the model from 130 to 230 GPa. For each value, the error (distance) between the predicted backbone tip position and actual tip position was found for the different tendon tensions combinations. The errors for each of the tendon tension combinations were summed, giving a total error value for a given *E*. The total errors are shown in Figure 8 over the examined range, with the value E=168 GPa minimizing the total error across the tendon tension range to 0.1422 m, thus this is the value used within the model for the rest of experimentation.

### 3.3 No End Effector Load Experiments

**FIGURE 9:**
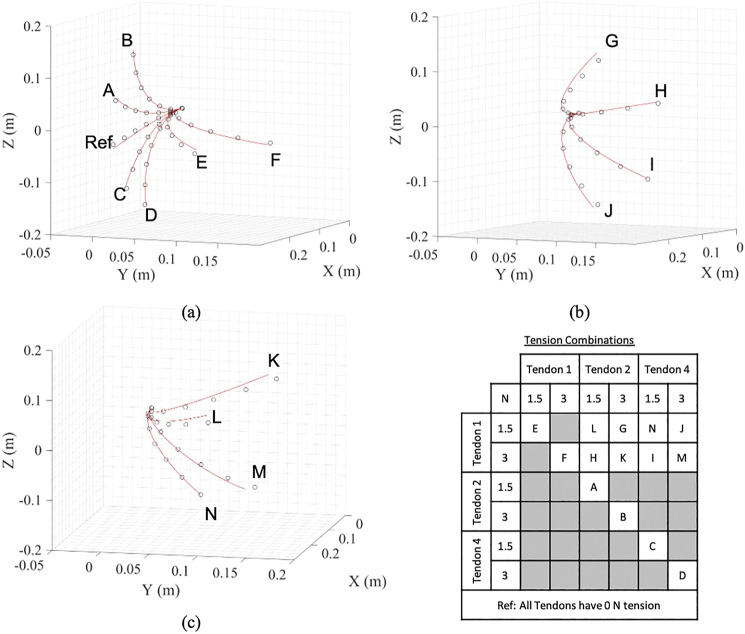
Predicted and realized backbone position for the **(A)** In-Plane, **(B)** Semi-Out-of-Plane, and **(C)** Out-of-Plane tendon region. The table summarizes the tension combinations for the different points. Red lines indicate model prediction and circles indicate the OptiTrack observations.

**FIGURE 10:**
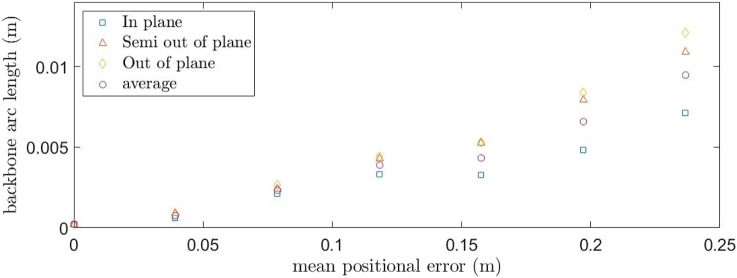
Mean positional error between the OptiTrack observations and model predictions for the three tension groups. The average result is included for overall comparison.

In this fist set of experiments the accuracy of the predictions from the derived model is investigated with no end effector. The updated Young’s Modulus from Section 3.2 and the friction countering approach from Section 3.1 are used. As stated above, it is assumed that the effect of the horizontal plane (tendons 1 and 3) would be symmetrical about 0 N. Each tension combination was measured three times and an average value was taken. Results showing the predicted and realized position of the backbone are given in Figure 9 for the three different regions defined within the tendon combinations; In-Plane, Semi-Out-of-Plane and Out-of-Plane test points. Figure 10 shows the mean positional error against the backbone arc length for the three different regions, at the sensor locations. In general there is an increase in the mean positional error as the backbone arc length increases. The In-Plane region has a significantly lower mean positional error with the Semi-Out-of-Plane region having a smaller error than the Out-of-Plane; this is expected as the backbone diverge from the fixed origin. This is due to the assumption that even for the Out-of-Plane bending, tendons are assumed to apply in plane moments to the end support disc.

Table 2 summarizes the mean positional tip location error together with the standard deviation for the three different regions. For the In-Plane region the error is 7.14 mm or 2.98% of the backbone length with a standard deviation of 3.21 mm. The Semi-Out-of-Plane and Out-of-Plane regions have an increased error of 10.9 and 12.1 mm or 4.56 and 5.05% of the backbone length with a standard deviation of 7.73 and 3.30 mm, respectively. This leads to an overall mean positional tip location error of 9.48 mm or 3.95% across all regions with a standard deviation of 4.99 mm.

**TABLE 2:** Mean positional tip location error together with the standard deviation for the three different regions.

Region	Error (mm)	Error (% of length)	Standard deviation (mm)
In-plane	7.14	2.98	3.21
Semi-out-of-plane	10.9	4.56	7.73
Out-of-Plane	12.1	5.05	3.30
All	9.48	3.95	4.99

### 3.4 Effect of End Effector Loads

In practice a continuum robot will have an end effector attached at the backbone tip location, therefore, investigations are carried out in which a vertical end load is acting on the tip of backbone. In this case the load was attached to the end support disk, where tendon 4 passes through it. The examined tensions in the different tendons are similar to those previously studied but with a reduce number of combinations of only 0 and 3 N for tendons 1, 2 and 4 with tendon 3 remaining slack. Four different loads were applied to the backbone tip of [0,96.14,189.92,293.32] N, with three repeats carried out and averaged.

**FIGURE 11:**
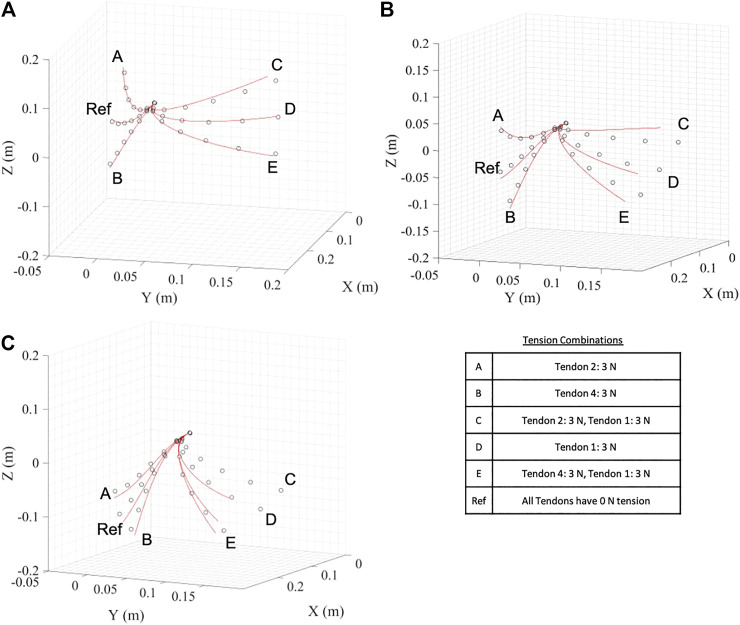
Predicted and realized backbone position for the **(A)** 0 N, **(B)** 96.14 N, and **(C)** 189.92 N tip load. The table summarizes the tension combinations for the different points. Red lines indicate model prediction and circles indicate the OptiTrack observations.

**FIGURE 12:**
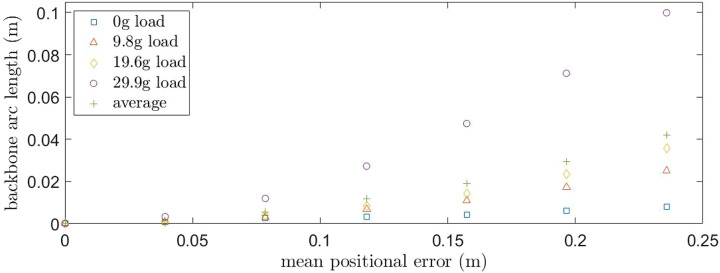
Mean positional error for different OptiTrack sensor backbone locations for increasing end load; 0, 96.14, 189.92, 293.32 N.

Figure 11 shows the predicted and realized backbone position for each load of the first three loads. The mean positional error between the predicted and realized positions along the backbone are given in Figure 12 for an increasing load on the backbone tip. Increasing the point load gives an increase in the mean positional error, with the tip error increasing significantly for the horizontal tensioned tendon cases; when tendon 1 has a non-zero tension. Table 3 gives the mean positional tip location error, together with the standard deviation, for increasing end load. Load 293.32 N results in the highest average positional tip error of 100 mm or 41.8% with a standard deviation of 73.3 mm, which is an order of magnitude larger than the smaller examined loads. The overall mean tip error for all cases is 42.3 mm or 17.63%, with a standard deviation of 73.33 mm.

**TABLE 3:** Mean positional tip location error together with the standard deviation for increasing end load.

End load (N)	Error (mm)	Error (% of length)	Standard deviation (mm)
0	7.88	3.28	3.72
96.14	25.28	10.53	15.38
189.92	35.75	14.89	31.16
293.32	100.36	41.81	132.86
All	42.32	17.63	73.33

## 4 Discussion

The mean tip error and standard deviation across all datasets with zero added mass was found to be 3.95% and 4.99 mm respectively, which compares favourably with similar continuum robot validation studies carried out by Chikhaoui et al. (2019) who achieved a total tip averages of 5.83% with a similar continuum robot validation study.

When investigating the effects of increasing or decreasing the tension to the desired tendon tension value, it was found that decreasing to the desired tension results in a significantly increased standard deviation of 7.77 and 9.43 mm for a tension of 3 N in tendon 4 and 2, respectively, whereas in the case of increasing the tension to the desired value, the maximum standard deviation is 2.94 and 2.59 mm, respectively. It is proposed that this situation arises because although the slack is removed in the case of over tensioning, the stiction/friction may cause a higher than desired tension remaining within the backbone past the base support disc. This higher energy state that the tendon has, is more likely to overcome the stiction/friction limit of the support disc and therefore more likely to randomly find a state of equilibrium. Therefore, the Teflon coated tendons used within the experimental facility still have significant friction effects. It would be advantageous to account for the effects of stiction/friction and slack within the model, as this would improve the repeatability of the continuum robot control as the desired tip position will most commonly be reached from an undeformed backbone position. The friction occurring between the tendons and the routing holes could be modeled as Coulomb/dry friction, and will effect the moment applied to the backbone tip Yuan et al. (2019), Gao et al. (2017). However, there will also be cases where decreasing the tension to the desired value is required and so reduction of the inherent tendon friction of the continuum robot should also be pursued. Nevertheless, there are no oscillations from the friction elements. It is assumed that the PID controller is acting to suppress any such phenomena. Moreover, the quasi-static nature of the movements ensures that any dynamic instabilities are quickly contained and then controlled.

Good agreement between the predicated and realized positions is achieved when there is a 0 N end load. However, for the cases when the end load is non-zero, there is an error between the predicted and realized results, which increases with end load. For tensions in only one plane, the errors are still relatively small, however when tendons in two planes are tensioned the error becomes larger. This discrepancy could be due to the backbone bending away from a single plane and causing the end load to produce an additional moment which is not replicated within the model. Therefore, it is important to know the center of mass of any tools to be applied to the end of a surgical robot as the tip position results can be significantly effected. Nevertheless, the mass of a surgical tool is likely to be small and as such the model would still be a reasonable representation of the real system.

## 5 Conclusion

In this work theoretical and experimental descriptions of an endoscopic continuum robot are examined. The representative model of the continuum robot configuration was based on Cosserat rod theory including gravitational effects, end loads and end moments produced by the tendons. A robust and efficient numerical solver was implement and validation was provided through the bespoke experimental facility. The experimental system actuates four tendons via motorized lead screw mechanisms that are instrumented load cells. To minimize the rise time of tendon tension, while avoiding steady state oscillations, a switching PID control methodology is implemented. An OptiTrack system (a contactless system) was used to determine the backbone deformation.

The tendon tension range, end load range together with the repeatability of the experimental facility were examined. A model parameter calibration gave an optimized backbone modulus of elasticity of 168 GPa, and model predictions gave the backbone tip position to within 9.48 mm of the experimental data, or 3.95% of the backbone length over the full tendon tension range examined. The In-Plane tendon tensions gave the theoretical and realized position being closer than the Out-of-Plane tendon tensions with calculated tip position errors of 2.98 and 5.05%, respectively. Despite the increased tendon slack introduced within the rig, increasing to the desired tension was significantly more repeatable than the decreasing method for high tendon tension cases with maximum recorded standard deviations of 2.94 and 9.43 mm, respectively. The unloaded model was observed to predict very similar backbone deformations to those from the experimental data. Results show that the model is sufficiently precise for practical implementations with predictions representing the backbone curvature and position well, showing a good compromise between computationally time and accuracy.

This investigation has provided an overview of the effect of the frictional effects of the tendons in the body of a continuum robot. Moreover, the proposed modeling and numerical approach in describing the kinematics of the robot have been validated and follow the experimental observations. The next step is to translate these findings into a smaller scale system, applying these outcomes to an endoscopic task together with evaluating the proposed approach with a system and corresponding controller that has a higher dynamic response. By taking into account the effect and behavior of the actuation tendons the precision and accuracy of the final system can be greatly improved. Moreover, the proposed methodology is applicable to multi-segmented continuum robots, since the model can be applied in a cascade fashion between segments with appropriate boundary conditions and minimum computational load.

## Data Availability

The raw data supporting the conclusions of this article will be made available by the authors, without undue reservation.
